# Association of Adjuvant Chemotherapy With Overall Survival in Patients With Early-Stage Breast Cancer and 21-Gene Recurrence Scores of 26 or Higher

**DOI:** 10.1001/jamanetworkopen.2020.3876

**Published:** 2020-05-04

**Authors:** Sung Jun Ma, Oluwadamilola T. Oladeru, Anurag K. Singh

**Affiliations:** 1 Department of Radiation Medicine, Roswell Park Comprehensive Cancer Center, Buffalo, New York; 2 Department of Radiation Oncology, Massachusetts General Hospital, Boston

## Abstract

This cohort study investigates the association of overall survival with receiving chemotherapy among patients with early-stage breast cancer and recurrence scores of 26 to 30 vs those with recurrence scores of 31 or greater.

## Introduction

A secondary analysis of the Trial Assigning Individualized Options for Treatment (TAILORx) randomized clinical trial^1^ demonstrated favorable distant metastasis–free survival among patients who received adjuvant chemoendocrine therapy for hormone receptor–positive, *ERBR2*-negative, axillary node–negative breast cancer and had 21-gene recurrence scores (RSs) of 26 and higher. However, 43% of patients had RSs between 26 and 30,^1^ and the chemotherapy benefit in this subgroup remains unclear. The omission of chemotherapy has not been analyzed prospectively in such a subgroup, and current treatment guidelines recommend the consideration of chemotherapy at the discretion of the clinician.^1,2^ Furthermore, although distant metastasis–free survival was previously shown to be worse among individuals with RSs of 31 or greater compared with those with RSs between 26 and 30, distant metastasis–free survival has not been validated in this patient cohort as a surrogate measure for overall survival (OS).^1^ To address this knowledge gap, we conducted a retrospective cohort study using a nationwide hospital-based cancer registry to investigate the association of OS with receiving chemotherapy and having an RS of 26 to 30 compared with having an RS of 31 or greater.

## Methods

Given that this study used a deidentified National Cancer Database (NCDB) database, institutional review board approval and patient consent were waived by the Roswell Park Comprehensive Cancer Center. Our report followed the Strengthening the Reporting of Observational Studies in Epidemiology (STROBE) reporting guideline.

The NCDB was queried for women patients diagnosed between 2010 and 2015 with hormone receptor–positive, *ERBR*-negative, axillary node–negative, T1-2N0 early-stage breast cancer with an RS of at least 26. We used the Kaplan-Meier method, log-rank test, and Cox multivariable analysis for OS analysis (eAppendix in the Supplement). Propensity score matching was performed using the nearest neighbor method in a 1:1 ratio without a replacement. The standardized difference of all variables was lower than 0.1, suggestive of adequate match.^3^ Analyses were performed with R version 3.6.1 (R Project for Statistical Computing). Statistical significance was set at *P* < .05, and all tests were 2-tailed.

## Results

A total of 17 197 patients with a median (interquartile range) age of 60 (52-67) years met our inclusion criteria. Of those, 12 741 patients (74.1%) received chemotherapy (4889 [38.4%] with RS 26-30; 7852 [61.6%] with RS ≥31) and 4456 patients (25.9%) did not receive chemotherapy (2993 [67.2%] with RS 26-30; 1463 [32.8%] with RS ≥31). The median (interquartile range) follow-up was 40.7 (23.4-60.8) months. On multivariable analysis adjusted for age, race, comorbidity burden, year of diagnosis, tumor size, surgery, and radiation, the addition of chemotherapy was associated with improved OS for all patients (hazard ratio [HR], 0.58; 95% CI, 0.50-0.67; *P* < .001), and regardless of receipt of chemotherapy, having an RS of 31 or greater was associated with worse mortality (HR, 1.75; 95% CI, 1.51-2.03; *P* < .001). Similar findings, favoring patients who received chemotherapy, were seen in 2081 matched pairs of patients with RSs of 26 to 30 who did and did not receive chemotherapy (HR, 0.70; 95% CI, 0.51-0.98; *P* = .04) (Figure and Table). Likewise, in 3278 matched pairs of patients who received chemotherapy and had RSs of 26 to 30 or of 31 or greater, having an RS of 31 or greater was associated with worse mortality (HR, 1.85; 95% CI, 1.42-2.40; *P* < .001) (Figure and Table). On interaction analysis, the magnitude of the chemotherapy benefit was comparable between patients with RSs of 26 to 30 (HR, 0.59; 95% CI, 0.46-0.76; *P* < .001) and RSs of 31 or greater (HR, 0.56; 95% CI, 0.46-0.68; *P* < .001; *P *for interaction = .70).

**Figure.**
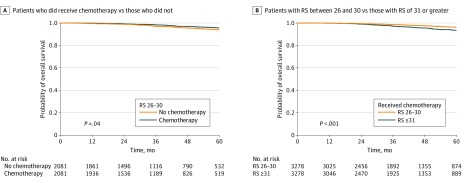
Kaplan-Meier Survival Curves After Matching Matching was performed for characteristics including treatment facility volume, age, race, education level, comorbidity score, year of diagnosis, histology, tumor grade, pathologic T staging, number of lymph nodes examined, hormone receptor status, type of surgery and radiation, surgical margin, radiation dose, postoperative readmissions, and duration of postoperative inpatient admission. RS indicates recurrence score.

**Table.** Baseline Characteristics for Matched Cohorts

Characteristic	Patients with RS 26-30, No. (%)		*P* value	Patients who received chemotherapy, No. (%)		*P* value
	No chemotherapy (n = 2081)	Chemotherapy (n = 2081)		RS 26-30 (n = 3278)	RS ≥31 (n = 3278)	
Facility volume						
Low	122 (5.9)	109 (5.2)	.42	129 (3.9)	125 (3.8)	.44
Intermediate	402 (19.3)	430 (20.7)		593 (18.1)	555 (16.9)	
High	1557 (74.8)	1542 (74.1)		2556 (78.0)	2598 (79.3)	
Age, y						
<65	1356 (65.2)	1375 (66.1)	.56	2612 (79.7)	2610 (79.6)	.98
≥65	725 (34.8)	706 (33.9)		666 (20.3)	668 (20.4)	
CDS						
0	1825 (87.7)	1780 (85.5)	.12	2967 (90.5)	2954 (90.1)	.86
1	215 (10.3)	250 (12.0)		284 (8.7)	295 (9.0)	
≥2	41 (2.0)	51 (2.5)		27 (0.8)	29 (0.9)	
Education						
More educated	1447 (69.5)	1388 (66.7)	.13	2311 (70.5)	2261 (69.0)	.19
Less educated	631 (30.3)	689 (33.1)		967 (29.5)	1017 (31.0)	
Not available	3 (0.1)	4 (0.2)		0	0	
Histology						
Ductal or lobular	2080 (99.9)	2081 (100)	>.99	3278 (100)	3277 (99.9)	>.99
Others	1 (0.1)	0		0	1 (0.1)	
Grade						
Well differentiated	258 (12.4)	254 (12.2)	.06	158 (4.8)	155 (4.7)	.98
Moderately differentiated	1119 (53.8)	1205 (57.9)		1508 (46)	1521 (46.4)	
Poorly differentiated	623 (29.9)	543 (26.1)		1531 (46.7)	1518 (46.3)	
Other differentiation	3 (0.1)	4 (0.2)		2 (0.1)	1 (0.1)	
Not available	78 (3.7)	75 (3.6)		79 (2.4)	83 (2.5)	
Race						
White	1820 (87.5)	1816 (87.3)	.18	2936 (89.6)	2925 (89.2)	.93
Black	178 (8.6)	157 (7.5)		222 (6.8)	229 (7.0)	
Other	69 (3.3)	93 (4.5)		107 (3.3)	113 (3.4)	
Not available	14 (0.7)	15 (0.7)		13 (0.4)	11 (0.3)	
Year						
2010-2012	845 (40.6)	836 (40.2)	.80	1385 (42.3)	1396 (42.6)	.80
2013-2015	1236 (59.4)	1245 (59.8)		1893 (57.7)	1882 (57.4)	
Pathologic T stage						
1	1627 (78.2)	1573 (75.6)	.05	2370 (72.3)	2414 (73.6)	.23
2	454 (21.8)	508 (24.4)		908 (27.7)	864 (26.4)	
Lymph nodes examined						
≤2	1105 (53.1)	1103 (53.0)	.54	1657 (50.5)	1692 (51.6)	.36
>2	968 (46.5)	974 (46.8)		1616 (49.3)	1584 (48.3)	
Not available	8 (0.4)	4 (0.2)		5 (0.2)	2 (0.1)	
Hormone receptor						
ER+/PR+	1645 (79.0)	1676 (80.5)	.31	2607 (79.5)	2600 (79.3)	.91
ER+/PR-	433 (20.8)	404 (19.4)		669 (20.4)	675 (20.6)	
ER−/PR+	3 (0.1)	1 (0.1)		2 (0.1)	3 (0.1)	
Surgery						
Lumpectomy	1493 (71.7)	1498 (72.0)	.89	2189 (66.8)	2189 (66.8)	>.99
Mastectomy	588 (28.3)	583 (28.0)		1088 (33.2)	1088 (33.2)	
Other	0	0		1 (0.1)	1 (0.1)	
Margin						
Negative	2028 (97.5)	2015 (96.8)	.48	3236 (98.7)	3246 (99.0)	.43
Positive	49 (2.4)	62 (3)		38 (1.2)	30 (0.9)	
Not available	4 (0.2)	4 (0.2)		4 (0.1)	2 (0.1)	
Radiation						
None	595 (28.6)	606 (29.1)	.87	1074 (32.8)	1083 (33.0)	.65
External beam	1380 (66.3)	1375 (66.1)		2112 (64.4)	2115 (64.5)	
Others	106 (5.1)	100 (4.8)		92 (2.8)	80 (2.4)	
Total						
Radiation dose, median (IQR), Gy	60 (52.4-61.2)	60.4 (52.6-62.0)	.08	60.4 (52.7-61.2)	60.4 (52.7-61.0)	.60
Readmission within 30 d						
None	1983 (95.3)	1973 (94.8)	.23	3105 (94.7)	3074 (93.8)	.09
Unplanned	30 (1.4)	20 (1.0)		42 (1.3)	45 (1.4)	
Planned	35 (1.7)	51 (2.5)		68 (2.1)	103 (3.1)	
Others	3 (0.1)	5 (0.2)		5 (0.2)	4 (0.1)	
Not available	30 (1.4)	32 (1.5)		58 (1.8)	52 (1.6)	
Postoperative inpatient duration, median (IQR), d	0 (0-1)	0 (0-1)	.44	0 (0-1)	0 (0-1)	.18

Abbreviations: CDS, Charlson-Deyo Comorbidity Score; ER, estrogen receptor; IQR, interquartile range; PR, progesterone receptor; RS, 21-gene recurrence score.

## Discussion

The magnitude of adjuvant chemotherapy benefit among patients with high risk (ie, RS ≥31) has previously been described.^4^ To our knowledge, this is the first study to report the association of adjuvant chemotherapy with improved OS in patients with breast cancer, with comparable magnitude between those with RSs of 26 to 30 and those with RSs of 31 or greater. Having an RS of 31 or greater remained independently associated with worse mortality compared with having an RS of 26 to 30 despite receiving chemotherapy, consistent with the secondary analysis of TAILORx.^1^ Pertinent variables, such as performance status, were unavailable in the NCDB, and lack of adjustment during matching could result in residual selection bias. In the propensity score matching analysis, postoperative readmissions and duration of postoperative inpatient admission were matched as proxy measures for postoperative complications and performance status prior to receiving adjuvant therapies.^5^ While we await further prospective studies, the data presented here may inform clinicians’ decisions for systemic therapy in patients with RSs of 26 to 30.

## Notes

Supplement. **eAppendix.** Supplementary Methods
