# Supplementary material for: Association of Adjuvant Chemotherapy With Overall Survival in Patients With Early-Stage Breast Cancer and 21-Gene Recurrence Scores of 26 or Higher
Source: JAMA Netw Open. 2020 May 4;3(5):e203876. doi: 10.1001/jamanetworkopen.2020.3876 (PMC7199111; doi:10.1001/jamanetworkopen.2020.3876)
Supplement: Supplement. — eAppendix. Supplementary Methods [file jamanetwopen-3-e203876-s001.pdf]

Supplementary Online Content

Ma SJ, Oladeru OT, Singh AK. Association of adjuvant chemotherapy with overall survival in patients with early stage breast cancer and 21-gene recurrence score of 26 or higher. *JAMA Netw Open*. 2020;3(5):e203876. doi:10.1001/jamanetworkopen.2020.3876

eAppendix. Supplementary Methods

This supplementary material has been provided by the authors to give readers additional information about their work.

© 2020 Ma SJ et al. *JAMA Network Open.*

eAppendix. Supplementary Methods

As our study utilized a de-identified NCDB database and institutional review board (IRB) approval was waived by Roswell Park Comprehensive Cancer Center. Our report follows the Strengthening the Reporting of Observational Studies in Epidemiology (STROBE) reporting guideline.

All missing values were coded as unknown for analysis. Pertinent variables such as specific comorbidities, performance status, type and duration of chemotherapy, toxicity, tumor recurrence, and breast cancer specific mortality, were not captured in the NCDB. The primary endpoint was overall survival (OS) defined as the time interval between diagnosis and the last follow-up or death.

Kaplan-Meier and log-rank tests were performed for survival analysis. Comparison of categorical and continuous variables were based on Fisher exact test and Mann-Whitney U test, respectively. Cox proportional hazard multivariable analysis (MVA) model was built based on all statistically significant variables from the Cox univariable analysis followed by a backward stepwise elimination. Variables of interest include age, race, Charlson-Deyo comorbidity score, education level, histology, grade, year of diagnosis, hormone receptor status, and types of treatments received.

The potential interaction between the use of chemotherapy and 21-gene recurrence score (RS) was evaluated by adding interaction terms to Cox proportional hazard MVA final model. The final model was re-assessed for RS 26-30 and RS>30 subgroups to compare the magnitude of the effect of chemotherapy.

Additionally, to address the selection bias, propensity score matching was performed based on all variables from the Cox proportional hazard MVA final model as well as other clinically relevant variables. Of note, regarding immortal time bias, 310 patients (1.8%) in our study either died or were lost to follow up within 6 months after diagnosis (n=142 without chemotherapy, n=168 with chemotherapy). We re-analyzed Cox multivariable analysis based on the remaining 16887 patients and our results show similar outcomes (RS>30 vs 26-30: HR 1.75, 95% CI 1.50-2.02, p<0.001; chemotherapy vs none: HR 0.59, 95% CI 0.51-0.69, p<0.001). Thus, it is unlikely that immortal time bias alone could lead to improved survival outcomes for patients who received chemotherapy.

All p values were two-sided and p values less than 0.05 were considered statistically significant.

© 2020 Ma SJ et al. *JAMA Network Open.*
